# Anticipation, agency and aging–conditions for making movement irresistible

**DOI:** 10.3389/fragi.2024.1380838

**Published:** 2024-08-14

**Authors:** Lise Amy Hansen, Wendy Keay-Bright, Felicia Nilsson, Heidi Wilson

**Affiliations:** ^1^ Institute of Design, The Oslo School of Architecture and Design, Oslo, Norway; ^2^ Cardiff School of Art and Design, Cardiff Metropolitan University, Cardiff, United Kingdom; ^3^ Cardiff School of Sport and Health Sciences, Cardiff Metropolitan University, Cardiff, United Kingdom

**Keywords:** artistic methods, irresistible movement, responsive technology, sensate movement, relational agency

## Abstract

This article describes an approach to developing and maintaining interpersonal agency through guided movement and responsive technologies. Making Movement Irresistible (MMI), considered conditions for developing a digital, online and wearable intervention that could make the act of movement irresistible for older residents in care, and encourage improvisational and social interactions. Working within a co-design framework, we combined making material objects and moving together as a method of examining the efficacy of human to human, and human to technology relationships to cultivate agency. Given that movement as performance is frequently not practiced or uncomfortable, we invited a variety of experts as our co-designers to notice the nuances of movement that interested them and to document these using drawing, writing and visuals. This documentation was gathered regularly in journals as the workshops progressed, leading to a coherent capture of data as it emerged. This data allowed us to attribute value to how simple actions could become a conduit for more ambitious, exploratory interactions. Our playful methods afforded the participation of co-designers, enabling us to situate our proposed intervention within a relational and social, rather than medical model, of ageing. Making movement do-able and relational, so that it can be shared and extended with a partner or carer, informed the idea to design a wearable device that could detect movement variability, resulting in a prototype, named emitts^®.^ The device makes use of the hand as way in to accessing whole body interaction. Our work with responsiveness of visual feedback avoided deterministic targets, as with no two movements being identical, the reported problem of compliance with repetitive tasks could be reduced. The technology foregrounded movement that was capricious and improvisational, offering new modes of artistic practice and engagement through play and performance. The case we describe highlights the importance of understanding the conditions that augment social interaction, rather than specifying design criteria for determining interaction. The longer-term health benefits of our intervention have yet to be measured, however, our collaboration has revealed how interpersonal agency emerges when we socially, aesthetically, and physiologically stimulate movement, making it irresistible where there may otherwise be resistance.

## 1 Introduction

What is it about repeated movement for the purpose of exercise, rehabilitation and “what the physio says,” that is unmotivating and, over time, an uphill struggle? It seems that approaching movement as exercise risks instrumentalising it and removing the potential agency residing in movement itself. Typically, carefully instructed movements may become medication-like, universal, problem-solving, and outcome-focused. And as we get older, we are increasingly encouraged to move with the basic rationale of improving our health through a programme of movements. Yet, within movement there is a potential of it being much more, and our explorations here promote a novel lens in a reframing of movement.

Making Movement Irresistible (MMI) proposes that we can approach movement as a creative and artistic material that may shape a social role: a material that can be performed together with or in relation to others. In reframing movement from exercise to a socially engaged playful relation, MMI sets out to create conditions for instigating and sustaining movement for older people in care homes–rather than relying on movements being specified and instructed. Our question concerns how we might encourage a process of discovery of one’s own movement and to try something new, overcoming insecurity and encouraging play and presence. In trying to answer this, the project took inspiration from artistic practices such as dance improvisation, dance scores and somatic practices (Noland, 2009; Burrows, 2010) as well as community arts and co-creation methods (Matarasso, 2019). Such practices acknowledge that movement and improvisation require “a type of cognition anchored in the body and situated in the relation with [dance] partners and space” (Ribeiro and Fonseca, 2011, p. 72). The use of artistic and improvisatory methods embraces a creative and participatory approach connected with individual transformation (Hanna, 2020). Our workshops involved a range of care provider experts in co-creative and explorative exercises over the course of a year where each workshop explored how to build on, and with, materials and relations by way of movement (see Table 1).

**TABLE 1:** Overview of workshops, objectives, participants, materials and activities with references to further workshop documentation.

Date	Objectives	Participants	Materials	Activities	Reference
Workshop One 26/04/2022 Duration 5 h	1. Who are we designing with/for? 2. What could make movement irresistible? 3. What are the barriers to engagement? 4. What opportunities are emerging?	12 primary care providers; dance artist, occupational therapist, physiotherapist, arts and health coordinator and NHS IT manager. 10 academic, technology and design staff	Socks, haberdashery, flip chart, crayons, markers, sticky dots, screen, projector, exercise books	Welcome and ethics consent and information Playful warm-up and creating sock puppets. Movement, sensation and participation activity. Personas, dot poll, innovation exhibition, wearable and biotech presentation	https://makingmovementirresistible.org/case-study-one/
Workshop Two 22/06/2022 Duration 5 h	1. What can technology add? 2. Could technology make movement irresistible? 3. What are the barriers to using tech? 4. What opportunities are emerging?	12 primary care providers; dance artist, occupational therapist, physiotherapist, arts and health coordinator and NHS IT manager. 10 academic, technology and design staff	Socks, haberdashery, flip chart, crayons, markers, microprocessors, light assets, screen, projector, exercise books	Making movement with puppets. Experiments with adding technology assets. Exercises with digital mirroring. Show and share of insights	https://makingmovementirresistible.org/case-study-two/
Workshop Three 03/11/2022 Duration 5 h	1. What movement do we desire? 2. How could we digitally respond to movement. 3. How do we make the whole experience irresistible and for whom?	9 primary care providers; dance artist, occupational therapist, physiotherapist, arts and health coordinator and NHS IT manager. 5 academic, technology and design staff	Low fidelity prototypes, box, card, haberdashery, flip chart, crayons, markers, microprocessors, light assets, screen, projector, exercise books	Group activity: Making prototype experiences such as day in the life, journey map and storytelling. Show and share of insights	https://makingmovementirresistible.org/case-study-three/
Workshop Four 18/01/2023 Duration 5 h	1. How do we provide meaningful interactions between facilitator/partner and older person? 2. What data will facilitators need? 3. How do we encourage sustained engagement?	7 primary care providers; dance artist, occupational therapist, physiotherapist, arts and health coordinator and NHS IT manager. 5 academic, technology and design staff	Medium fidelity wearable prototypes, flip chart, crayons, markers, screen, projector, exercise books	Group activity: Designing the looks and feel. Ease of use and engagements before use. What may make this movement engagement different and engage health practitioners? Online and in person	https://makingmovementirresistible.org/case-study-four/s
Workshop Five 02/05/2023 Duration 5 h	1. Who are we designing with/for? 2. What could make movement irresistible? 3. What are the barriers to engagement? 4. What opportunities are emerging?	8 primary care providers; dance artist, occupational therapist, physiotherapist, arts and health coordinator and NHS IT manager. 7 academic, technology and design staff	Medium fidelity wearable prototypes, flip chart, crayons, markers, screen, projector, exercise books	New personas and people mapping. Can-do movements. Digital Playtime. Journey mapping. Concept of emits ^®^ consolidated	https://wahwn.cymru/knowledge-bank/-making-movement-irresistible-mmi

During the workshops our physiotherapy and occupational therapy partners reported that they face a significant challenge with patient adherence to a prescribed exercise programme. They advised us that despite being given instructions on how to perform their prescribed movements, older people were frequently unmotivated to practise the recommended exercises. Research indicates that adherence is influenced by low baseline levels of physical activity, poor self-efficacy, depression, anxiety, helplessness, poor social support, greater number of perceived barriers to exercise and increased pain levels during exercise (Kåringen et al., 2011; Rowsell et al., 2022; Room et al., 2023). Older people are particularly at risk of low adherence and are likely to have multiple and long-term conditions for which prescribed exercise is a treatment option (Room et al., 2017). Addressing this gap in behaviour, we foregrounded movement as material with which we could connect the mover, carer and technology through certain conditions and by addressing the agency in movement itself.

The range and severity of movement impairments in older people needs a broader, relational (peer-to-peer), less diagnostic and more motivational approach to exercise (Peek et al., 2016). Findings from our initial research indicated a need for imaginative techniques that can offer choice and agency within exercise routines. For example, using metaphors such as “move your arm like a swan” rather than an instruction for lifting your arm (Butt, 2017, p. 343). Recent research shows that dementia is not a hindrance for explorative learning (Ingebrand, 2023). What MMI has aimed to do is to explore movement beyond its everyday function, by cultivating aesthetic capabilities.

## 2 Artistic, sensate, and aesthetic practices

Improvisation is a form of creative practice whereby a set of rules or parameters may frame new movements and meanings (Burrows, 2010) and thus invites a person or performer to be in contact with their individual aesthetic sensibilities. These sensibilities are tied to perception, which is at the core of exploration, learning and creative imagination; as we improvise the moment, we cease to know what is going to happen (Tuffnell & Crickmay, 1993). For movement, the felt experience of moving–kinaesthesia: “allows us to correct recursively, refine, and experiment with the practices we have learned” (Noland, 2009, p. 4). In improvisation the interaction itself “gives us access to the intentions and emotions of the other as well as to a field of shared interactively created meaning” (Kronsted and Gallagher, 2021., p 39).

We approached the research project from the premise that sparking the imagination was key for engagement, and consequently sought to probe these aesthetic sensibilities in greater depth, seeking what may be identified as a flow state where movement is “a self-justifying experience; it is, by definition, an end in itself” (Csikszentmihalyi, 2014, p. 250). From our previous research we have learned to value the perceptual qualities of sensation, attention, repetition, feedback, feedforward, and connection, as conditions that may be harnessed in a design concept (Hansen et al., 2017; Wilson, 2017; Sumner et al., 2019; Keay-Bright et al., 2021). Typically, these aesthetic and kinaesthetic experiences are ephemeral and intangible. However, the impact of bringing these elements into consciousness is known to improve agency (Reynolds and Reason, 2012) and improve kinaesthetic empathy - that is the access to the perception of one’s own or others another’s kinaesthetic experience (Parviainen, 2003).

### 2.1 Anticipation and immediacy

Whilst words may take time to access and compose, the moving body affords a gestural immediacy, expressing a multitude of meanings that may be implicitly understood (Tversky, 2019). Yet even with the immediacy of instruction and presence of mind, there is evidence to suggest that a period to prepare, and to stimulate ahead of moving “may be beneficial in preparing for movement readiness and thus result in better motor execution by the patient, and hence better rehabilitation results” (Crasta et al., 2018, p. 111). As the aim was to encourage movement, we understood that the relation and preparation and context of an exercise or an engagement is central in how it is to be performed. This can, in part, be described as entrainment: “a spatiotemporal coordination resulting from rhythmic responsiveness to a perceived rhythmic signal” (McNeill, 1995, p. 3) and can be valued as a social practice (Phillips-Silver et al., 2010) that may be developed and refined (Phillips-Silver and Keller, 2012). It has been found that an injured brain can indeed access rhythmic entrainment mechanisms (Crasta et al., 2018). Research in entrainment points to the foundations for how people typically engage in a reciprocal manner: “as we converse, we increasingly use each other’s words and gestures” (Tversky, 2019, p. 111) and in people, coordination quickly turns into cooperation (Ibid).

### 2.2 Guided movements as a mode of engagement

Guided movement suggests that movements created in relationship with another person, as in empathetic facilitation, invite interaction where sociality and connection provide purpose and motivation (Christensen et al., 2021). To support an understanding of movement in care home contexts, dance improvisatory practices such as those introduced by post-modern dancers in the US and the UK in the 1970s provide a useful reference point (Mackrell, 1992). Whilst an improvised dance performance event may be scaffolded by a “score”, a set of rules or parameters which frame an improvisation (Burrows, 2010), the movement content also unfolds as dancers respond moment by moment to each other’s dancing bodies and other stimuli. Works are not set, and the dance vocabulary is not usually predetermined: “For each moment in improvisation, the dancer reacts to her own current kinesthetic unfolding in order to make the next movement” (Kronsted and Gallagher, 2021, p 41). The principles behind contact improvisation are also relevant here, in that this is a partner dance which progresses by attending to your partner through points of physical contact and weight sharing and acting on one’s own pull of gravity (Paxton, 1975). Moving away from theatre dance, principles that guide community dance practice are helpful in defining an ethical basis for dance interactions with non-professional dancers (People Dancing, 2024). In this approach, the movement is created through the perception and relations to others, which, in turn, creates a renewed sense of self. Research has shown that intersubjective relations are key in dance-health practice “in enabling the dance artists, acting as guides, to facilitate a heightened awareness of somatic and subjective lived body experience” (Hanna, 2020, p. ii).

### 2.3 Designing engagements

Aesthetically-led processes can bolster engagement in the crafting, shaping and making of materials towards a design - whether an object, event or practice (Hallnäs, 2011). Movement as creative and artistic material challenges the notion that materiality is only physical. Movement is performed by our physical bodies and manifested in every instance, yet it is this temporality that also means that it is forever disappearing (Phelan, 2003). This ephemeral aspect of movement is essential for how we typically see and handle movement. Still, we also have an experience of the lasting effect a movement can have - from a moment or over time (Hansen and Lyster, 2023). When we focus on movement as a creative, aesthetic material, it has implications for the understanding of aesthetics. In interaction design, aesthetics has moved its focus from appearance to interaction (Djajadiningrat et al., 2000), recognising our perception of aesthetics in any one moment as dynamic and changeable. Theories for interaction aesthetics focus on its temporal nature, whereby concepts such as pliability, rhythm, dramaturgical structure, and fluency play an important role in conceptualising aesthetics (Lowgren, 2009, p. 5). In art, the concept of Relational Aesthetics (Bourriaud, 1998) summarises this changed view on aesthetics, with an emphasis on the temporal and interpersonal interaction, which happens in between people when the stage is set. The physical, static form of the work itself - the stage - is of less importance. It is the situation that is created and what that does (its performative potential) rather than what it is (the staging) that becomes interesting.

### 2.4 Exploring technology through dynamic, sensory engagement

In dance improvisation the perception of movement is shaped by “an ongoing cycle of acting on affordances and being acted upon. Each movement brings out new possibilities that the dancer must engage to keep the participatory sensemaking activity going” (Kronsted and Gallagher, 2021, p.43). Making use of this dynamic opens up a possibility to leverage individual agency and relational movement, as it frames how a perception of movement may continuously be interpreted in relation to its preceding movement.

The benefit of a wearable device is that sensors providing feedback may be designed to provide a unique focus on detecting and responding to particular movements (Keay-Bright, 2011), thus feeding into an entrainment practice–a relational synchronisation of new movements. With age there is typically a physical reduction of movement possibilities, yet Jung has shown that directed self-awareness may expand the scope of personal data through the use of mobile and wearable applications that draw attention to how people may “notice, express, question, and respond to their felt senses” (Jung, 2023, p.30). Phillips-Silver et al. propose that for entrainment to emerge, three central aspects are required: “to perceive stimuli as rhythmic, to produce periodic stimuli, and to integrate the two using sensory feedback” (2010, p. 10). There is a need to order and amplify the feedback from movement and that the feedback should be direct, clear and playful (Keay-Bright et al., 2021), for example, using a range of light effects that can draw attention to, and enhance visual feedback by exaggerating aspects of the movement itself (Hansen et al., 2017).

In dance and dance improvisation dancers and instructors often work with mental images to get the right kind and quality of movement, which could be prompts such as “imagine moving in honey” (Christensen et al., 2021, p. 17). A focus on the materialising of feedback in an aesthetic manner was a central feature in this project, and a variety of feedback modes were explored–such as sound, light, colour, line, and shape.

Houston and Gillette (2022) offer a map of soft skills that surround effective practice when leading dance in a community context, which includes empathy, dealing with uncertainty, cooperation, taking care of others, flexibility and adaptability, managing information, patience and appreciating difference. They suggest that these skills “encourage individual and personal approaches to working relations and social encounters” (Houston and Gillette, 2022, na).

**FIGURE 1:**
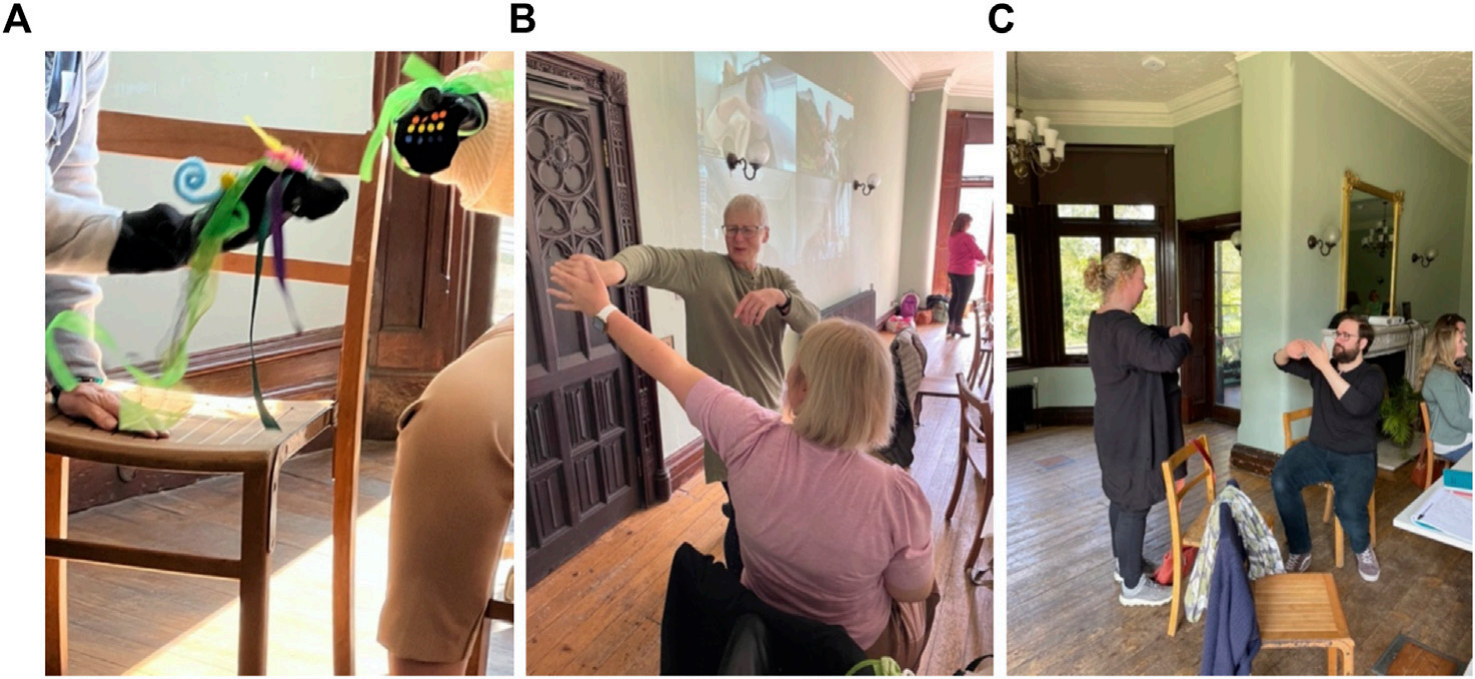
**(A)** Exploring movement beyond its everyday function through cultivating playful social interactions. **(B)**: Sensory attention to touch and relationship allows movement to emerge organically in an unfolding “conversation.” **(C)**: using mirroring to prompt moving together and to overcome uncertainty.

These soft skills were explored during our workshops, beginning with a mode of dance improvisation that offered inexperienced dancers the same starting point as more confident dance practitioners (see Figure 1). Using movement to build connection with another person requires willingness. Openness, curiosity and creativity, and the suspension of judgement of self and others, which was vital in this arts-informed project.

As action moulds perception (Tversky, 2019), all actions may, in turn, enable new actions, if reframed as a source of engagement or possible relation. The motivation to move then, may be reframed in the form of experimental movement, imagination and play in relationship with another person and enhanced through responsive technology. Seeing movement as the unfolding of one movement into another, and as a self-justifying experience, may assist this entrainment of the senses. Our experiments with camera-projector-screen-based responsive technology, further enabled us to explore a digital materialisation of movement that in turn, enabled play - as immediate and visceral.

## 3 Materials and methods

The Making Movement Irresistible project required the academic and practical study of movement from a wide range of disciplinary perspectives and brought together an academic team with expertise in biometric science, stroke and dementia research, interactive and material arts, dance and music technology. Our participants were experts from clinical practice and arts intervention, including dance artists/practitioners, occupational health and physiotherapists and a range of activity providers working in the care sector. This partnership has been achieved through the known networks of the academic team, whose research prioritises participatory and inclusive methods within iterative cycles of design and implementation. The team has significant practical experience of including people with a range of age-related disabilities, such as stroke, dementia and complex needs, in research and a track record for creating evidence-based interventions that develop self-efficacy through human-to-human interaction and technology (e.g., Adams et al., 2015; Hansen et al., 2017; Wilson, 2017; Sumner et al., 2019; Buckley et al., 2020; Treadaway et al., 2020; Keay-Bright et al., 2021).

Our method for involving arts and health partners as collaborators in envisioning a movement intervention was a series of five co-design workshops that ran over a period of 1 year. The workshops were structured around five core questions: who, what, why, where, and when and together the workshops addressed the overarching question, “what makes movement irresistible?” Posed as questions, these five workshops enabled us to examine the conditions that connect us to our moving bodies, for example, the people, materials, activities, settings, and participatory infrastructures that may affect the desire to move. Three workshops were hosted at a local conference venue, a Victorian listed building with gardens, and two were held at the university design studios. Participants who were unable to travel joined online. Whilst this was challenging at first, as the community of participants became more familiar with one another, we started to design the workshops with online activity as a feature rather than an alternative.

### 3.1 Research design

In addition to the core questions that pertained to the conditions that make movement irresistible, the workshops also explored how movement could contribute to co-design. For example, how making physical artefacts and performing everyday actions - such as mirroring and waving–could, gently and inconspicuously, prompt moving together. The ease of this activity supported an experience of designing that became increasingly creative. The aim was to investigate whether participants felt a sense of belonging and agency when the sensation of movement was brought into consciousness. Such an approach was valuable, as we were interested in tapping into the kinaesthetic and empathic potential of movement. In each workshop participants took notes in individual exercise books. Exercises, discussions and presentations were filmed. The principal researcher summarised the resulting material from each workshop. This was sent out to all participants to enable the planning of next steps, and informed the iterative, qualitive thematic analysis driving the development forward.

As the workshops progressed toward the “where,” relationships became the most vital condition for making movement irresistible. However, as none of our workshops were held in a care home due to the pressures on staff, the project invited professionals who would normally deliver activities in care homes to participate in a dedicated workshop space. Here, they shared the experience of working with older people, who had had a lifetime of being with others and who now relied on staff, each other, and visitors for company. Moving together was described as a key motivator for engagement (Houston, 2019). The intention was to shift movement from being instrumentalised and normative, and to optimise moving as empathic, social and creative. The kinaesthetic and empathic potential of movement was an important factor in the preparation of the aesthetic aspects of workshop activities, such as using fabric and feathers, and including new sensorially interesting materials as we developed prototypes. This materialisation was designed to allow for a directness, clarity and playfulness of feedback, whether it was a signalling hand or digital visuals and the anticipation of new feedback.

## 4 Summary of workshops

**FIGURE 2:**
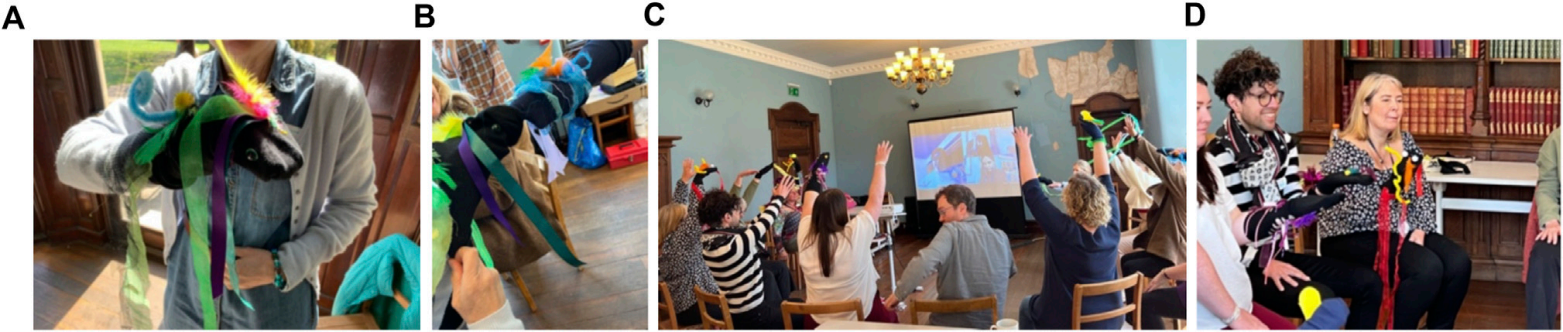
**(A, B)** Participants’ exercise books forming an essential part of qualitative data collection. **(C)**: Exercise where a web of ribbon re-defines the space as a playground for imaginative movement responses, whereby the situation is created and what that does, becomes an interesting outcome. **(D)**: Adding “light” as a sensory component to a sock-puppet using simple electronics.

The first workshop posed the questions: who are we designing for, and what might make movement irresistible for this audience? As an introduction to the project, to each other and the sensation of movement participants were given a range of haberdashery materials and a plain black sock, from which they were invited to create a puppet. When worn on the hand, the puppet fulfilled the role of “alter-ego” and prompted people to meet, greet and move together in response to gentle, guided suggestions given by the experienced dance practitioner from the academic team. Beginning with gentle hand movements, we were able to gradually explore the liminal space between the anticipation of moving and the curiosity that sparks more conscious, playful and intentional action. Actions and gestures change thought, both those we perform ourselves and those we see in others (Tversky, 2019). In particular, hands have agency in how they can make up gestures through an “immediacy, precision, and congruence of their meanings.” (Tversky and Jamalian, 2021, p. 772). Our exercises played with this idea of the hand as a gateway for connection to others as well as a connection to moving the rest of one’s body (see Figure 2).

As participants became more relaxed with each other, we introduced camera-based digital mirroring opportunities using software created for an earlier project (Keay-Bright, 2011; Keay-Bright, 2014; Kontogeorgakopoulos, Weschler, Keay-Bright, 2014) and some face-to-face mirroring experiments with hand gestures. Participants also created simple personas, which sparked a discussion on the value of relationships rather than diagnostic representations of individuals.

Workshop two posed the question: what could we be creating? The objective was to scope the technological, artistic and biometric potential of MMI in relation to the findings from workshop one. The workshop consisted of four activities: 1. Making movement, 2. Digital movement, 3. Making more movement and 4. Moving on.

**FIGURE 3:**
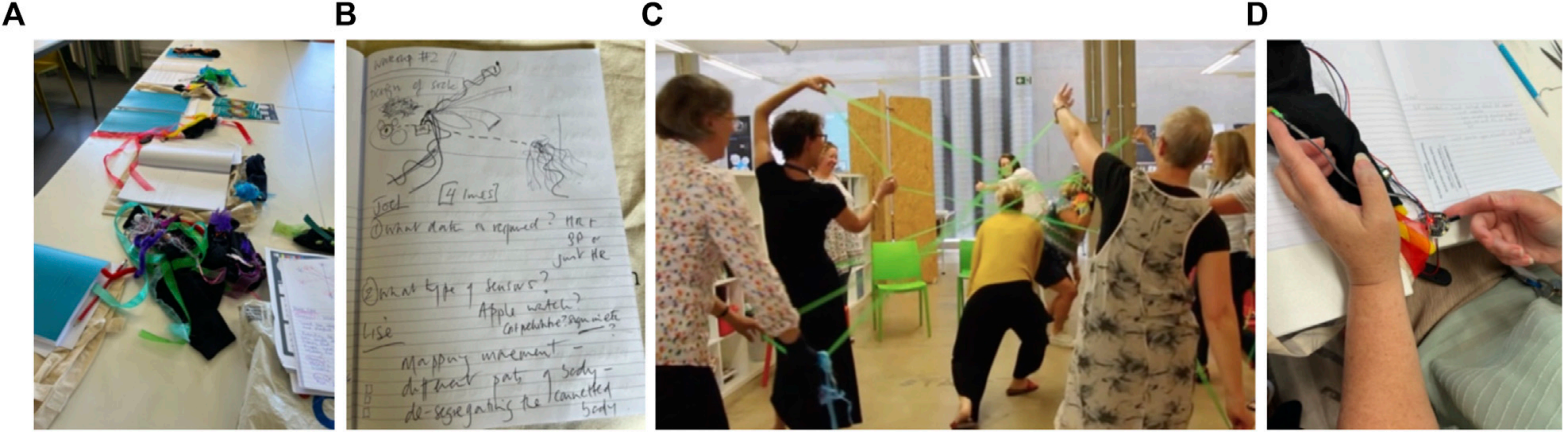
**(A, B)** Participants’ exercise books forming an essential part of qualitative data collection. **(C)**: Exercise where a web of ribbon re-defines the space as a playground for imaginative movement responses, whereby the situation is created and what that does, becomes an interesting outcome. **(D)**: Adding “light” as a sensory component to a sock-puppet using simple electronics.

Participants were given a range of light generating electronic assets that could be hand sewn into the sock puppets. This provided an opportunity to observe whether electronic feedback could feel playful and irresistible - or if it would induce fear and rejection. As a creative tool, the electronics engendered a myriad of ideas for improving manual dexterity and functionality, demonstrating a fine line between generating movement drills and expressive, improvisatory actions. Unsurprisingly, the potential to capture, log and visualise heart rate variability as a non-invasive feature of interaction met with interest from health professionals. More significant was the idea that visually arresting heart-rate data could be a prompt for improvisatory movement, whereby both health and creative objectives could coincide (see Figure 3).

**FIGURE 4:**
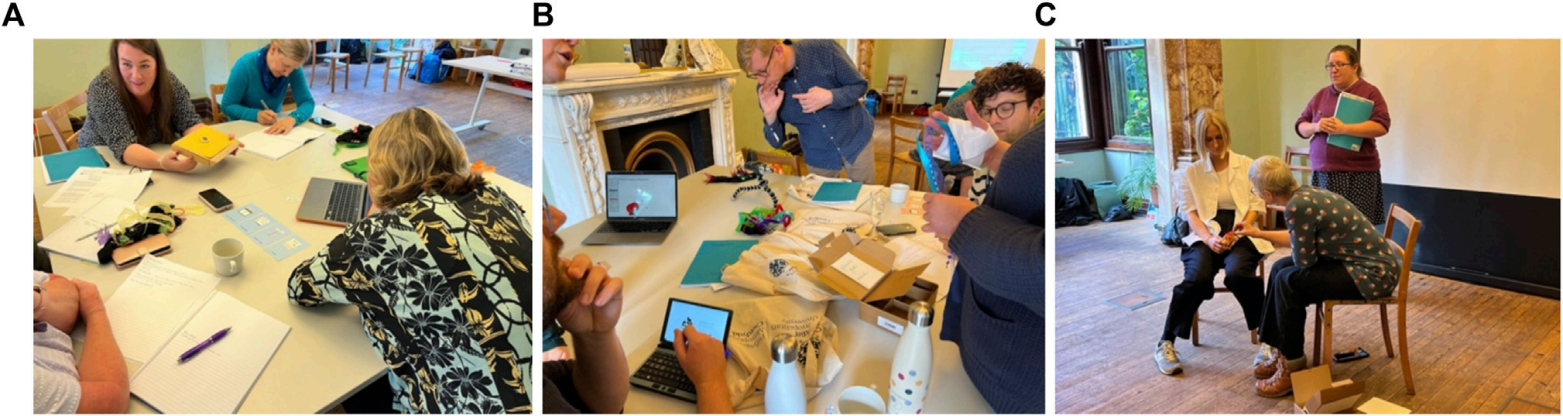
**(A)** Journey mapping exercise of conditions that connect us to our moving bodies such as people, settings, aesthetic materials and playful movement invitations. **(B)**: Exploring a low-fidelity prototype to see amplification of movement contributions through aesthetically pleasing digital feedback (such as sound, colour, light, line and shape). **(C)**: Acting out inter-personal agency as social, empathic and aesthetic movement interactions.

The third workshop introduced early concepts for making movement irresistible as a more holistic experience. A key question at this stage was what are we offering and how do we encourage people to engage? An important finding from workshop two was the need to fully understand each of the stages that contribute to making moving irresistible - from the anticipation of moving, through to the curiosity induced by movement sensations, to the agency of being able to experience movement as communicative and creative in partnership with another mover. Working with a paper prototype concept for a customisable, wearable mitt with electronic assets and screen-based feedback, participants were invited, in three teams, to build three different scenarios and sketch out the stages of interaction. Using the prototype as a prop to generate stories, each team acted out scenarios for how the concept could be implemented and explored within a variety of practice situations (see Figure 4).

**FIGURE 5:**
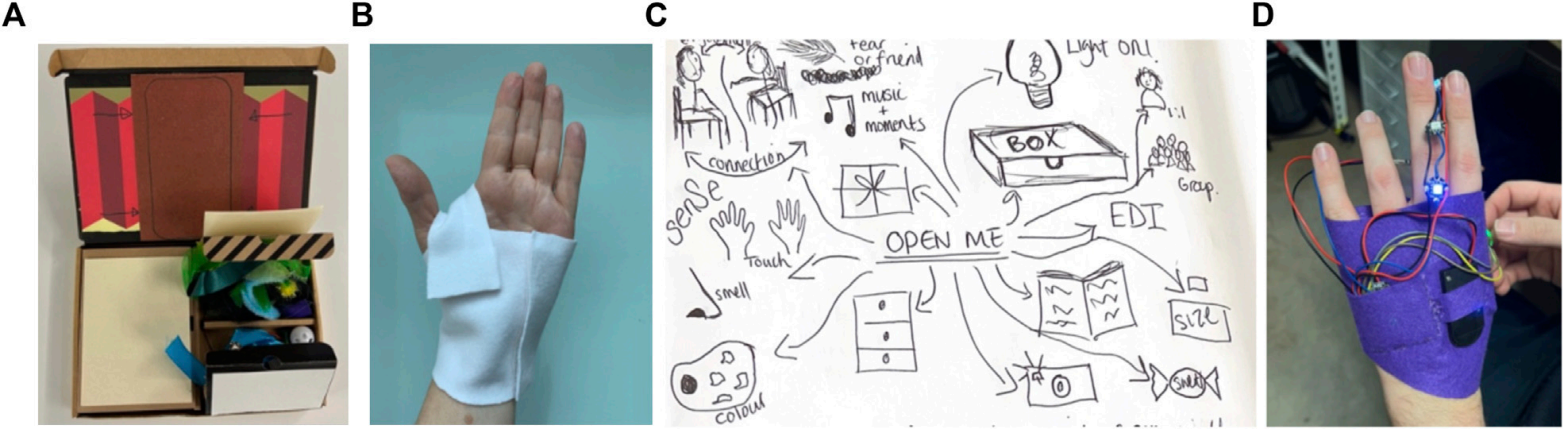
**(A, B)** Medium fidelity prototype of a presentation box containing a wearable mitt with optional sensory accessories. **(C)**: Relationship Map showing how the “mitt” might reach the intended users. **(D)**: Medium fidelity prototype “mitt” linking lights to motion sensors where lights turn on and off in response to the tip and tilt of the hand.

The fourth workshop focused on sites and timelines, taking the idea of implementation as a cue for further experimentation, plus a relationship map which identified who would be responsible for ensuring the concept reached the intended users (beneficiaries). The question of aesthetics was foregrounded in the discussion as a range of visual effects were offered. Ideas emerging from light-response interactions prompted experiments on the theme of bioluminescence, which was proposed as an opportunity to create iconic, visually coherent feedback sensations. A more refined version of the prototype was presented, which, although crude in the visual aesthetic, became a tool for envisioning how the hand and mitt could generate bioluminescent effects (see Figure 5).

**FIGURE 6:**
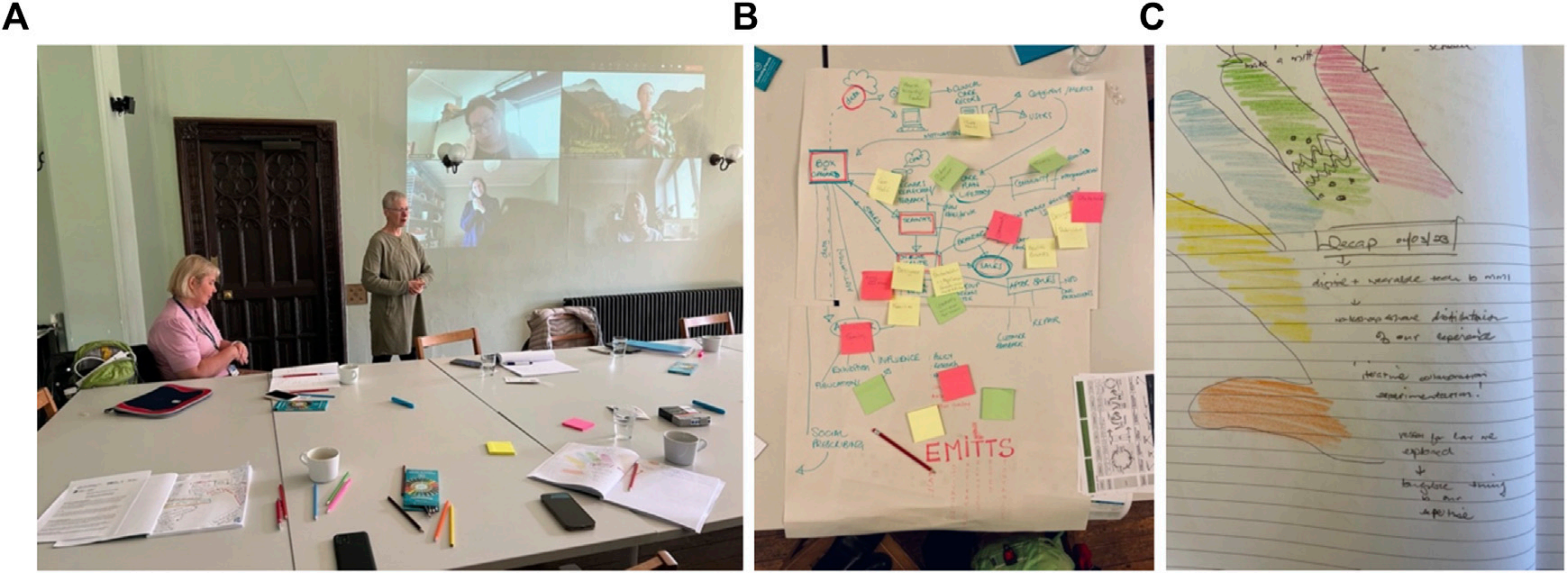
**(A)** Participants both online and at Insole Court being guided through a sensory mapping of their hands. **(B. C)**: participants’ diagrams of stages required to introduce emitts^®^ into a potential care environment.

The fifth workshop revisited the questions of: who, what, why, where, and when, in relation to what we had discovered about, “what makes movement irresistible”. A short film was shown, capturing key movement moments and conversations from the previous workshops. Participants were invited to reflect on what had changed in their thinking on each of the questions and asked to add anything new that came to mind. A more refined prototype was then demonstrated, and participants were given time to play and make further suggestions on implementation. In three teams, the participants created large maps and flow diagrams, capturing the stages required to introduce such a technology into a care environment (see Figure 6).

## 5 Resulting discussions

The Making Movement Irresistible arts, health and technology project described above, has given us leverage to explore what it means to move in conversation with another person. Through our workshops, ideas emerged that prompted us to take note of the nuances of these conversations as we forged new relationships with our partners. Over the course of the five workshops, we were able to become more attuned to the conditions for initiating and maintaining relational movement. The various conditions we have noticed and explored over the workshop have taught us about i) creating engagements, ii) movement for engagement and iii) movement for relational agency. A discussion of these and the role of the technologies follows in the sections below.

### 5.1 Creating engagements

The invitation to engage in creative and expressive movement can be met with uncertainty, discomfort and resistance. The predominant concept of dance also implies a required level of physical and cognitive skill, as a further barrier to engagement. Making and moving with prototypes can divert attention away from the pressure to perform and attune people to engage with the sensate aspects of moving.

#### 5.1.1 Materialising props

The workshops identified the need for a tangible, intimate, observable and shareable device for accessing the proprioceptive sensation of movement, and halfway through the workshops, a prototype wearable mitt was created. The mitt became a materialisation of the hand as a gateway to communication; simple waving exercises, tapping, clenching and other “everyday” movements could become an interesting means to explore larger bodily connections, and as a starting point for further movement. By acknowledging that movement is relational, it was important to include the developing prototype in movement explorations as a ‘partner’ which could shift, shape, alter and augment a movement conversation between people. Compliance could be facilitated by the attention of a partner through mutual explorations of performed movements that are enhanced and detailed by way of engaging wearable technology.

#### 5.1.2 Encountering uncertainty and discomfort

Creating conditions for movement to emerge includes gaining motivation and confidence, whilst building the capacity for movement within the mover. In this way, personal movement practice and agency are closely interlinked. The aim is to develop and support an embodied agency - a person’s possibilities and capacities for action and expression. We used qualitative thematic analysis (Cresswell and Poth, 2016) whereby reflections and participant data from each of the workshops led us to focus on the hand as a gateway to movement interactions. The hand is vital for gesture and, “just as the actions of our hands are tools that alter the world, the actions of our hands are tools that alter minds, our own and those of others” (Tverksy and Jamalian, 2021, p. 772).

Exploring improvised creative movement can initially create feelings of discomfort, uncertainty and self-consciousness. This is something participants in the workshop expressed. Yet, the process of overcoming this discomfort also leaves you with a feeling of having learnt something new about yourself. This challenge was explored precisely by introducing artistic processes to participants who were not artists. Providing an enjoyable experience may in itself reduce agitation, increase sociability and promote interaction (Christensen et al., 2021). Absorption in a pleasant activity may then reduce experiences of failure and frustration in both “‘patient” and therapist if the prescribed exercises could evoke aesthetically motivated sensations.

Such thresholds and learning steps are found in trying out new practices. New practices demand uncomfortable learning thresholds. Tolerance of these experiences varies from person to person. Workshops engaging our senses in new ways are challenging. These methods allowed us to uncover what could be problematic and what could be gainfully explored further together. When facing older age and needing to expand or recover a movement range, we must look at the “unconscious patterning, unexamined inhibition, and corporeal reservations that are only apparent when challenged by new kinaesthetics” (Downey, 2010, p. S27).

#### 5.1.3 Flow and agency

Pleasurable experiences of movement flow facilitated through a shared MMI interaction may support persistence to overcome feelings of uncertainty and discomfort. What we learned throughout the workshops relates to trust and agency, and the value of revisiting reflections and insights learned: “gaining bodily skills requires more than ‘knowledge,” involving changes in physiology, perception, comportment, and behaviour patterns in unsystematic, diverse modes’ (Downey, 2010, p. S22). Experiments with visual feedback on mobile devices were used to capture and replay movement as a simple graphic form, explorations of space and temporality were introduced using connecting ribbons and hand clapping games. Together these activities initiated new sensations, new reactions, responses and perceptions.

This technology, however, was introduced, staged, played out and performed in a social setting–with others, whether as carers, therapists, experts, or “audience,” as movement is always perceived and performed in relation to its context. Kendon has also pointed out that for signs “to be transmitted, they must be seen” (Kendon, 1995, p. 116). Over the course of the workshops, we expanded our focus from a low-fidelity prototype towards the possibility of both a wearable mitt and screen-based interactive technology. We found that the role of the facilitator became crucial over time, and the prototype could become part of this relationship. This allowed workshop participants to anticipate an interaction, and consequently have the agency to influence a triadic relation (older person-partner-technology) where shared movement creates a space of possibility.

When seeking to understand such conditions leading to an irresistibility of movement, the phenomenological experience of “flow” is worthy of attention. Sustaining one’s effort in continuing to move was part of the challenge identified: “The creation of specialist bodily knowledge occurs through the ability to sense and enact shifts in temporalities and spatialities” (Carter et al., 2022, p. 248). Flow is characterised by attention to the activity which brackets out other distractions to the extent that people report a sense of agency as actions and awareness merge and perception of time is altered (Csikszentmihalyi, 2014). During the workshops we experimented with several exercises that encouraged participants to move and play individually. As their confidence and subsequently, their ideas developed, participants created a variety of scenarios, which they acted out using role-play to tease out the relationship between staff, therapists, families and older people. An example scenario was using the mitt prototype to engage a stroke patient, in which a dancer used feathers, colours and touch to draw attention to sensations and to find ways to make it easy to move together. The potential for creating experiences of “shared flow” as an outcome of an MMI interaction is a tantalising possibility.

### 5.2 Movement as engagement

We found that agency of movement required an understanding of the physical space, giving permission and attention, and allowing for sensate feedback in play.

#### 5.2.1 A temporal, physical taking of space

Reframing space as creative and performative, for example, for dance, makes for a liminal space just beyond the everyday. Schechner (2017) takes an ethnographic perspective on such performative events, suggesting seven functions: a vehicle for fostering community; marking or changing identity; creating something beautiful; as performance; to heal, entertain and to persuade. In a private space witnessed only by yourself and your dancing partner, Schechner’s characterisations of functional performance may be useful, giving licence to play, subverting restricting social conventions and resisting a regression to “normalcy” in support of relationship building and acceptance of difference. In a care home context, dedicated time for one-to-one interactions can be difficult, however making opportunities for your personal space to be reframed to your “stage” affords a temporal, physical and aesthetic environment where you may perform to the limits of your available space.

#### 5.2.2 Permission to perform

Permission to express yourself can be seen as an outcome of being given attention and active listening. Research on the value of active listening in medical consultations shows a potential therapeutic value of affective physician-patient communication and positive relationship building (Fassaert et al., 2007). Fassaert et al. identify features of active listening, many of which are reliant on non-verbal communication such as an open and interested attitude, giving full attention and not being distracted, being relaxed, giving time and including comfortable pauses. Parallels can be drawn between the use of active listening in medical consultations with doctors and the attention required in dance improvisation.

Non-verbal understanding is communicated through gestures, facial expressions, eye contact, occasional physical contact and the use of paralinguistic cues, that is, communication beyond linguistics and language. In its ambition to make movement irresistible, this project explored an approach to developing meaningful movement that expands on health motivation and cognitive abilities. Our findings point to the complexities in understanding how people in care homes may choose or know to move, or the immense challenges when one cannot move as one wishes. Finding ways to cope (Noland, 2009) is important and being given a space and ways to accept, face and explore challenges are vital, and as we found, part of the conditions for initiating meaningful movement.

#### 5.2.3 Attention to joy

When a facilitator, such as a physiotherapist or a dancer, expresses curiosity they can act as a magnet for joint exploration of movement. The choreographer Burrows suggests that in dance improvisation “even a little is enough. Are you enjoying it? It should not be a chore” (2010, p. 54). By providing a focus on sensations and gentle movement possibilities with the hands, however small, it may lead to an unfolding movement “conversation”. Events or structures within such a conversation may include copy, contrast, call and response, touch, or with some people hand-over-hand supported movement. Through the hands as a point of contact the facilitator can “listen” and respond to their partner. An additional use of props and curious prompts which offer texture, colour, shape, sound or light, for example, can provoke a movement response and joyful joint exploration within a multi-sensory experience: “Understanding the senses in an ecological manner means ensuring the connections between a body and its environs, the body-in-the-world affirmed *via* sensory interactions and the information generated, remains embedded in the contexts in which sensory information is produced” (Carter et al., 2022, p. 242). Using material can also ease inhibition through deflecting attention from oneself to the object of curiosity. In our workshops, participants who were self-conscious of moving creatively found that the use of a personalised “sock puppet” eased them into a creative frame of mind and freed them to move with less inhibition. Making the puppets into individual characters also facilitated socialisation through play. With loss of inhibition, the movement range and complexity increased. Some people then chose to discard the puppet for reasons of greater freedom in movement and a more immediate “me to you” relationship without mediation.

Houston and Gillette (2022) recognise that a positive experience of joint creativity and joyful interaction allows us to build awareness of each other and to see each other differently. This, in itself, can be transformative. We found that framing movement as irresistible and magical, new and worthwhile, allowed us to identify the conditions necessary to initiate, draw attention to, and sustain movement, however small and however self-referential. It allowed for an anarchic space to celebrate deviation particularly for those people with cognitive differences or dementia.

### 5.3 Movement for relational agency

We found that giving or sharing the agency over new and sustained movements was part of fostering a positive relationship between partners and it is vital to be offered the motivation and curiosity to engage with one’s own movements in conversation with others.

#### 5.3.1 A shared agency in moving anew

For people living with dementia or sensory loss in a care context in Wales, UK, there is a commitment to offering “the right for your care to be delivered in a positive and caring way and staff should take time to get to know you” (Herklots, 2022). Anticipation of a rewarding and fulfilling encounter in which you feel valued, seen and to which you can contribute in a way that is meaningful to you, is predicated on a relationship of trust and respect. Our workshops drew attention to the need to design for care and activity providers who were not dance practitioners, they may even be a relation, friend or other health professional. A sense of achievement can be supported by the facilitator if they are attuned to the individual and able to modulate the demands of the interaction: too challenging and they disengage, too easy and they get bored and distracted.

Preston-Dunlop attests that the dance and the dancer are indistinguishable where “dancing is feeling-thinking-sensing-doing with imagination” (1998, p. 55). Attention to the qualities of movement promotes an attention to aesthetics: “in dancing with aesthetic attitude you focus on the attributes of the thing you are making, on the form and rhythm of the movement you are creating, on the relationships in space and time that your movement gives rise to” (Preston-Dunlop, 1998, p. 41). The movement exercises tried out in the workshops could be seen as similar to those experienced in functional everyday activities or rehabilitation exercises. What makes the movement unfamiliar and therefore curious is its relocation as a shared aesthetic experience.

Although it takes time to internalise and embody cultural practices (Mauss, 1973) our bodies are continuously changing and adapting - even within a limited age framing. Teixeira et al. (2021) describe how older people may be able to handle and engage with technology. However, in our view, technology is not the facilitator of the interaction, it is a partner in a creative conversation, much as the sock puppet became a conduit for a performative partnership for codesign participants. It can be liberating to interact with technology, as ‘nobody’ is watching or judging. When moving with the mitt, we found an opportunity for joint attention, and the beginnings of shared empathy as partners acted and reacted to each other.

#### 5.3.2 Designing for authenticity

Houston (2017) defines agency within inclusive dance as “a degree of self-determination of events by individuals that gives them the power to interact with and to act upon events positively”. Authentic, shared communication is central to experiencing agency as it allows us to express our social, physical, practical needs. This approach to developing autonomy within a shared space is at the heart of interventions such as Intensive Interaction (Nind and Hewett, 2012). As a social communication approach, Intensive Interaction has improved outcomes for young people in educational and care contexts with severe learning disabilities and autism. Although not “outcome driven,” intense positive reinforcement allows agency to arise as an intrinsic process of meaningful engagement. Our experiments with camera-projector-screen-based responsive technology, enabled us to materialise the flow of movement in digital form, which, in turn, enabled play - as immediate and visceral. The combined experience of positive feedback and the curiosity to discover more of one’s potential to create magic affords a similar intensity, as each movement stimulates the next. The partner’s role in the interaction is less about correcting and more about promoting a continuous somatic reflection-in-action as a duet. Over repeated interactions, a repertoire of movements can be developed and enjoyed. This may occur through instigating movement or indeed through the rejection or acceptance of an improvisation opener offered by a facilitator. Jung recognises such improvisation as an intentional component of a design brief: “Reflection through focusing on felt senses has been shown to lead to alternative design propositions and to induce self-knowledge beyond tracking past actions” (Jung, 2023, p. 15). Designing authentic movement interventions will require more uncertainty, more involvement, and more variation, yet such a development may also provide real value in enjoying movement and living well.

## 6 Conclusion

The transdisciplinary group of partners who engaged in researching and co-designing Making Movement Irresistible came together to develop a greater understanding of how to engage and experiment with movement, framed as an explorative study funded by the Arts Council of Wales and Cwn Taf Morganwg University Regional Partnership Board. These experienced professionals worked together to offer a novel lens for reframing movement of older people in care home settings.

Through this collaboration the project brought particular aesthetic aspects into focus–visuals, materials, and movement - by drawing attention to the manner in which we respond to a hand wave, or the touch of a textured material or the pace and sensation of a breath. We invited movement tasks that promoted curiosity, complexity and playful interactions. This increased the variety of body actions, relational dynamics and spatial dimensions that we were able to explore. The preliminary prototype intervention is called emitts^®^, which is a pair of fingerless mitts with responsive light emitting components and bluetooth connectivity to generate screen effects. In response to the feedback that the whole experience of anticipating and exploring with emitts must be irresistible, we have created bespoke packaging that is designed to make using emitts as stress-free and playful as possible. The prototype is showing potential for enticing older people to re-discover the joy of moving, yet the prototype is yet to be tested and developed further. As such, the longer term impact of what it may offer in a care setting has yet to be fully evaluated. The prototype has been developed to encourage the freedom to explore, as it enables a heightened sensation whereby new movements draw attention to the capability of the hand and the rest of the body may follow - as one is able. Whilst a screen could place the attention outside of the body–as we are so easily mesmerised by dynamic, interactive visuals on a screen–emitts draw attention back to the body rather than to remove it–that could allow for a heightened sensation of movement.

We have reframed movement and the conditions that could allow for a movement practice that has agency and value in variation. Our main contribution is conceptual in that we offer a set of conditions for reframing movement, as social, sensate and relational in support of health and rehabilitation goals. These new effects and sensations may give a renewed appreciation of movement, beyond tasks and health goals. The removal of instruction can make it easier to be curious about the smallest of movements and lead to a variety of extensions. With the methods and the prototype described in this article, we wish to contribute to a knowledge transfer between the arts and health disciplines. Movement belongs to both, is studied by both and is a concern for both. The expert groups were able to find unity in their expertise within this frame. The iterative workshops, and the reflection and analysis that took place between each, informed how one could develop technology towards increasing movement, access and agency. To this end, the notion of the irresistible was crucial to initiating movement. When an ageing body contracts rather than expands, prescribed movements may become too instructed, too foreign and simply, too tedious.

In this articulation of an arts and movement project, our intention has been to make the case for applying rigorous, artistic processes in health, care and wellbeing contexts. Deliberately using movement as method, development and outcome, we have been able to draw out conditions for creating curiosity, engagement with and joy of one’s aged body.

## Data Availability

The data presented in this article are available on the MMI online repository: http://makingmovementirresistible.org.
